# Embodiment and agency in a digital world

**DOI:** 10.3389/fpsyg.2024.1392949

**Published:** 2024-09-06

**Authors:** Nivedita Gangopadhyay, Alois Pichler

**Affiliations:** ^1^University Library, University of Bergen, Bergen, Norway; ^2^Department of Philosophy, University of Bergen, Bergen, Norway

**Keywords:** agency, cognition, embodiment, non-embodied agency, digital technology, interaction

## Abstract

We are agents and our agency is often best characterized in terms of embodied cognition. However, this is not to deny that there are cognitively significant ways of agentive engagement with the world that may be described without referring to our embodiment. In this paper we shall focus on the interplay between embodied agency and non-embodied agency or agency that may not be straightforwardly described in terms of embodied cognition in the context of interaction with digital technologies. In recent years a lot of our daily lives are coupled to the world via digital technologies. Yet how to understand the nature and evolution of our agency in the context of interacting with daily digital technologies is an open question. We propose to address this question by focusing on whether the steady development of digital technologies in our daily lives challenges the view that embodied agency is the de facto way of robustly engaging with the world and if embodied cognition is challenged then what is taking its place in scenarios where it was once dominant.

## 1 Introduction

Debates in contemporary philosophy of mind and cognitive science are structured around two main rival views of cognition and the mind in general. There is the view of the mind and cognition as abstract symbol manipulation or manipulation of abstract mental representations. This view drawing from the works of authors like Alan Turing (1912–1954), and subsequently developed more systematically with the emergence of modern-day cognitive science, builds broadly on some ideas typically associated with René Descartes (1596–1650), most notably the assumption that the mind and the body are two separate entities. While it is problematic to describe views that consider cognition to be abstract symbol manipulation as views that straightforwardly support a mind-body ontological dualism, they nonetheless adhere to a general principle where cognitive tasks can be defined and specified without reference to the embodiment of the cognitive agent (e.g. Mahon and Caramazza, 2008; Adams and Aizawa, 2009; Spaulding, 2011). Let us call this group of theories that claim that an agent's cognitive engagement can be defined and specified without necessarily drawing upon the agent's embodiment in any significant way, “non-embodied cognitive agency” (NECA). This group of theory stands in contrast to views of embodied cognition. Embodied cognition is a diverse group of theories that broadly trace their origin back to the phenomenological school of thought (e.g. Merleau-Ponty, 1945/2002; Gallagher and Zahavi, 2008/2021; Shapiro, 2019). Embodied cognition views also occasionally identify with 4E cognition views where cognition is seen as embodied, enacted, extended and embedded (e.g. Newen et al., 2018). For our present purposes, we shall focus on embodied cognition understood as the view that an agent's cognitive engagement significantly draws on the agent's bodily capacities of interaction both in terms of ontogenesis of a cognitive skill and in terms of real-time deployment of the skill. Let us call embodied cognition views of agentive engagement with the world “embodied cognitive agency” (ECA).

In this paper we revisit the debate between NECA and ECA in the context of our interaction with digital technology. The philosophical background or conceptual basis of digital devices such as computers and smart-phones is a computational view of cognition. Building on the idea of a Turing Machine (Church, 1937) as a simple abstract computational device, computationalism came to be consolidated for the further development of theories of cognition as theories of information-processing in David Marr's seminal work *Vision: A Computational Investigation into the Human Representation and Processing of Visual Information* (Marr, 1982). In this book, David Marr lays down the fundamental information-processing/computational principle underlying the development of an information-processing device. The principle states that the computational level of description, the level where the most important cognitive processing is described, may be, and indeed *should* be, defined independently of the level of hardware implementation, i.e. the embodiment level. Thus, modern computational devices are built upon a conceptual separation of cognition and embodiment.

As digital devices become increasingly interactive, ubiquitous and unavoidable in our daily lives, we are in a position to revisit the debate between NECA and ECA as a debate that is no longer simply centered on how we should understand human cognition but rather as a debate about how we should understand human cognition as it plays out in robust interactions with digital information-processing devices fundamentally built on the idea of separation of cognition from embodiment. The rethinking of the debate becomes all the more urgent if we consider that these ubiquitous information-processing systems have entered a, most likely irreversible, phase of technological development where they will increasingly implement artificial intelligence (AI) capabilities but not necessarily embodied cognition. In the context of discussions of embodied cognition, AI capabilities are at best weakly based on embodiment because the most embodied context of training for AI is deep neural network and embodied cognition theorists have long argued that embodied minds are not simply their brains (e.g. Noë, 2009).

Our goal in this paper is to focus on agentive engagement as an aspect of cognition and explore how to best think of *our* agentive engagement i.e. the agentive engagement of embodied beings, in the context of interaction with digital devices that are built on a fundamental conceptual separation of cognition and embodiment. We do not aim to argue for either NECA or ECA as a more acceptable view of cognition (and the mind) but simply lay bare some emerging issues in the debate between these views in the context of technological development. In the following we formulate the discussions around the notion of embodied agency (e.g. Merleau-Ponty, 1945/2002; Gallagher and Zahavi, 2008/2021; Segundo-Ortin and Heras-Escribano, 2023) and a notion of non-embodied (mental) agency that builds on a critique of embodied cognition (e.g. Block, 2001; Mahon and Caramazza, 2008; Adams and Aizawa, 2009; Aizawa, 2015; McClelland, 2020).

### 1.1 Embodied and non-embodied interaction

In our daily lives we interact with the world, including with other people, via digital technologies in significant and extensive ways. The smart phone, for example, has become almost ubiquitous in our lives, as have the use of computers and other digital devices. In addition, there are technologies that remain hidden to many of us but which nonetheless influence, and may even decide over, our lives in important ways, for example, the steadily increasing use of AI across the digital world.

In at least some of these interactions via digital technologies, we exercise our agency.^1^ Yet the degree of embodied engagement required while interacting as agents via digital technologies is arguably reduced when compared to more non-digital ways of interacting. Tasks that once required robust embodied engagement can now be performed with the touch of a button on a screen or typing on a screen. Let us start the discussion with an example.

Consider the case of AI chatbots for medical advice. Let us say that you are worried about a pain in your body and your doctor's office has the practice of screening patients first through chatbots before they get an appointment with the doctor. Let us also assume that the chatbot in this case is a fairly advanced one and asks relevant questions and gives helpful answers. In your chat session with the chatbot you communicate via typing. You do your best to communicate the nature and severity of your pain via words with an artificial agent who has been fed and trained on large datasets and is capable of meaningful linguistic communication for screening purposes. Yet interacting with the chatbot via digital interfaces does not draw on your embodied skills of interaction in a non-trivial way as would have been the case if you were to interact face-to-face with the doctor and explain the nature of your pain. The lack of embodied engagement while explaining to another cognitive agent the subjective nature of your pain may also present a few challenges for you. These challenges do not necessarily invalidate the expertise or purpose of the chatbot but rather create a different way of performing a cognitive task which in this case is communicating about something as subjective and fundamentally phenomenal as pain.

First, you may have to communicate everything you feel via words. There are no bodily expressions involved in this interaction. If you were meeting the doctor in person, it is reasonable to expect that a significant amount of your discomfort could have been communicated via embodied expressions such as grimaces or grunts or other embodied cues that doctors are trained to spot. These embodied expressions structuring your interaction with the doctor would have communicated *how* you felt the pain, or your subjective experience, without you having to use so many words as you perhaps would with the chatbot. Moreover, as social agents we have an implicit expectation that our embodied meaning-making is understood by our interaction partner.^2^ So you would have the expectation that the doctor will understand your mental state(s) in terms of their own bodily knowledge of interaction (e.g. Gallese, 2005; Gallagher, 2020). In the case of the doctor, the cognitive task of communicating the deeply subjective experience of pain is highly likely to be scaffolded by embodied interactions. Yet, in the case of the chatbot you will have to perform the same cognitive task i.e. convene the same subjective information to an artificial agent, but the artificial agent arguably has never experienced pain and cannot draw on any of the embodied interactions that a doctor could have used to scaffold the exchange of such information.

Second, when you are interacting with the chatbot and trying to express as clearly as you can in words the nature of your pain, you may experience a sense of distancing from your body. It is as if you have to take an objective stance on your body, reflect about the nature of the pain and then put in words the feeling of your body. However, in the doctor's office you could express the pain *through* your body in terms of embodied expressions. To borrow a term from phenomenological philosophy, your “lived body” (Merleau-Ponty, 1945/2002) would have been an integral part of your cognitive task of communicating in the doctor's office. But it is not necessarily a part of communication with an artificial agent via digital media. Thus in the latter scenario, one exercises cognitive agency without necessarily drawing upon embodied skills of interaction. This is in contrast to non-digital face-to-face communication with the doctor where one would draw upon embodied interaction to perform the cognitive task of conveying information about one's illness. Thus in case of the chatbot-interaction there is clearly less embodied interaction between two agents and the cognitive task of exchange of information between them occurs, at least apparently, without any significant role played by embodiment. Let us call such potentially less embodied ways of interacting “non-embodied” interaction.^3^^,^^4^

Non-embodied interaction via digital technologies may be a matter of degree with some technological interfaces strongly focusing on “virtual” embodiment such as virtual bodies while others seem to function without *apparently* engaging embodied skills in any significant sense, for example interfaces that operate only via linguistic communication. In either end of the spectrum, embodied skills of interaction as we know and experience in comparable non-digital interaction are modified. At one end of the spectrum our embodied agency is transformed, and occasionally augmented, in virtual realities and at the other end of the spectrum, e.g. texting via smart phones, they are apparently almost completely muted. In this complex and evolving scenario of technological adaptations, what is obvious is that our embodied agency as played out in similar non-digital contexts of interaction does not get transferred unchanged in digital contexts of interaction. Our goal in this paper is to explore some emerging issues in the context of interaction between humans as fundamentally embodied cognitive agents with devices that are fundamentally built on a principle of separation between cognition and embodiment. Thus we shall explore issues such as—does the steady development of digital technologies in our daily lives challenge the view that embodied agency is the de facto way of robust agentive engagement with the world, including with other people? Moreover, if digital technology's takeover of our everyday lives is challenging our embodied ways of interacting with the world and performing cognitive tasks, then what is replacing embodied cognitive agency in scenarios where it was once dominant?

We do not wish to focus on what type of object is involved as the digital device under consideration, e.g. whether it is a mobile phone or a computer, but rather on a cognitive task. Thus, the chatbot in our previous example could be on a mobile device or on a computer. What is important for our discussions is the task it is performing, which in this case is collecting information to act as an interaction partner in a communicative context and eventually make a decision about what has been communicated to it. Our focus on cognitive tasks instead of on the type of object does not, however, imply that the latter is irrelevant to the discussion. It is just that we choose to focus on cognitive tasks for the purposes of this paper. Of course, there may be instances where the task and the type of material interface are strongly intertwined. If such is the case, we will mention it in our discussions. In the following section we elaborate on the importance of the body in cognition as advocated by embodied cognition theories with roots in phenomenological philosophy and go on to situate the body in the context of cognitive tasks performed by and with digital devices.

## 2 Embodiment as experiential unity

The importance of the body in our everyday experience is extensively discussed by the phenomenological tradition in philosophy (e.g. Husserl, 1929; Merleau-Ponty, 1945/2002; Heidegger, 1975). Gallagher and Zahavi (2008/2021) succinctly summarize the role of the body in experience in the following words, “...the body is considered a constitutive or transcendental principle, precisely because it is involved in the very possibility of experience. It is deeply implicated in our relation to the world, in our relation to others, and in our self-relation, and its analysis consequently proves crucial for our understanding of the mind-world relation, for our understanding of the relation between self and other, and for our understanding of the mind-body relation” (Gallagher and Zahavi, 2008/2021, p. 135).

Phenomenological discussions of the body emphasize a peculiar duality of the body (e.g. Zahavi, 2001). The body may be given to one in experience as an interiority or as a dimension of sensing, and it can also be given as an exteriority, as for example when visually or tactually exploring one's own body. Alternating between these two experiences of the body is in most cases effortless and automatic. The reversibility between the interiority and the exteriority confirms that they are two-sides of an experiential unity. This peculiarity of the body becomes especially relevant in the context of intersubjectivity or when we understand other bodies as other minds (e.g. Zahavi, 2001). In the phenomenological tradition, it is common to think that by apprehending my own embodied subjectivity^5^ as given as a dimension of sensing, I come to apprehend other perceived bodies as being similar loci of embodied subjectivity (e.g. Merleau-Ponty, 1945/2002).

A crucial insight from the phenomenologists' discussions of embodiment for our current purposes is that multi-agent interaction and social cognition is fundamentally built on our embodied capacities of multisensory integration. Merleau-Ponty developed a detailed account of our understanding of other minds in terms of a tight multisensory coupling, e.g. between the modalities of vision, touch and motor cognition. Reflections on the duality of the body, i.e. as an interiority and an exteriority, led Merleau-Ponty to propose that vision and touch are two sides of the same modality (e.g. Merleau-Ponty, 1945/2002, 1969). Again, the duality of the body that is given as an interiority or as a dimension of sensing and as an exteriority when we explore our own bodies, also establishes the coupling between the sensory modalities like vision, touch and motor cognition. Meaningful experiences arise from sensory information when we bodily interact with the world either in terms of overt motoric engagement and/or in terms of covert deployment of motor routines. This is especially the case for social cognition and intersubjective construction of meaning. For example, when one sees another's hand reaching out to grab a cup then one's visual information is processed by coupling it to the motor routine that one oneself would have performed if one were to reach for the cup (e.g. Gallese and Goldman, 1998; Gallese, 2005). The motor routine in this case is implicitly or covertly deployed which means that one does not actually execute the action of reaching for the cup oneself but runs an offline simulation of the motor routine to make sense of the visual perception of seeing another reach for the cup. By such a coupling of vision and motor cognition one comes to ascribe an agentive intention, e.g. “she wants to drink from the cup,” to the other person. This type of understanding of another agent's intentions is often described in the literature as embodied understanding (e.g. Gallagher, 2005; Gallagher and Zahavi, 2008/2021; Fuchs, 2013) since it draws on one's own agentive capacities of embodied engagement with the world. Since digital devices are typically used in multi-agent contexts of interaction, insights about the role and nature of embodied cognition in agentive interaction are particularly relevant for understanding how humans as embodied cognitive agents interact with other agents in a digital context.

### 2.1 Embodiment and interaction in a digital context

The detailed, phenomenological discussions of the duality of the body and the role of embodied cognition in agentive interaction, which is further established by empirical evidence of visuo-motor coupling mechanisms in social understanding (e.g. Gallese and Goldman, 1998; Gallese, 2005), make a strong case for the lived body as the key to successful agentive interaction. The lived body, the locus of multisensory integration, serves a crucial meaning-making function in that it structures our fundamental interactions with the world, especially with other agents.^6^

Coming back to our discussion of digital media, we shall focus on how some types of interactions unfold in and with digital media, and on how our lived body figures in these interactions. In the preceding section we noted that the way our embodied agency plays out in non-digital contexts of interaction does not necessarily get transferred unchanged to digital contexts of interaction. Let us start by thinking of digital interaction as a broad spectrum. At one end of the spectrum of digital interaction we have fairly “non-embodied” forms of interaction where, for example, we communicate with a chatbot via a textbox on a screen. At the other end of the spectrum, we have highly immersive scenarios, for example, games played in highly immersive platforms as well as future hyper-real, super immersive virtual worlds.

For practical purposes, the kind of digital interaction that makes up most of our daily life lies somewhere between these two above-mentioned extremes of simply typing and extensive immersion. Our smartphones and digital interfaces like computer screens are extensively used in daily interaction with others.^7^ From the perspective of multi-agent interactions, there are certain technical and physical limitations to our embodied agentive interactions via these interfaces. For example, one may be interacting with an AI program that has a simple visualization on a screen, e.g. our medical chatbot. In this case it is reasonable to maintain that one is interacting with an agent that lacks “embodiment” in any significant sense of the term. Yet, it does not necessarily negatively affect either one's own performance of a cognitive task or one's ascription of intelligent cognitive agency to these non-embodied bots (e.g. Kim and Im, 2023). As Kim and Im (2023) write, “The most current technological products are virtual disembodied agents such as Apple's Siri and Amazon's Alexa. Although these agents are disembodied, humans tend to seek interpersonal relationships and social connections through communications and interactions (Fan et al., 2017)….” In an in-depth discussion of the affective experience of being connected to the Internet through digital devices, Krueger and Osler (2019) explore the unique properties of the Internet that allows it to scaffold affectivity in what the authors term “techno-social niches.” Although the authors reserve the discussion of artificial intelligence for further research, their analyses of affect generation and regulation via digital devices strongly points toward conceptualizing artificial agents as rich interpersonal interaction partners that are able to engage a fundamental aspect of human consciousness, namely, its affective dimension. However, such interaction may not necessarily lead to positive affective experience for the interacting human partner as, for example, Candiotto (2022) argues. Candiotto discusses the example of a young person living alone in a smart flat with an artificial agent and explores the phenomenology of complicated affective relations a human agent may experience with an artificial agent and in the context of “hyperconnectivity” that is constant online connectivity. This person may come to experience what Candiotto terms “extended loneliness” which is a “...new type of loneliness…experienced in the user's extension through technological devices” and it is a “...complex affective experience of both lacking and longing for relationships while being connected to artificial agents and people online” (Candiotto, 2022). Thus, Candiotto's discussions reveal that robust agentive properties may be attributed to artificial agents even in the complete absence of any significant embodiment of these agents, and that interactions with these non-embodied agents have the potential to deeply impact the affective lives of human interaction partners.

In the context of our present discussions the question arises—in these interactions with non-embodied agents, who are accepted by humans as interaction partners, does one draw on one's own capacities of embodied interaction to communicate with these agents? It is not immediately obvious that these interactions directly recruit the human agent's embodied cognition of the lived body. As we noted in the preceding discussions, the sense of the lived body is based on multisensory integration and is needed to make sense of the other agent's *perceived body* as the locus of similar multisensory integration. But in cases like the simple medical chatbot, as well as Siri and Alexa there is simply no multisensory input in any significant sense. Any visual input that may be relevant for ascribing agency to these agents is either too weak or non-existent. There is no need for perception of intentional behavior or of bodily movement in interacting with these agents. Thus, as the NECA view states, ascription of agency and acceptance of these agents as interacting partners does not directly draw upon our embodied cognition skills. In these and similar cases of digital interaction one may contend that embodied cognition is not our de facto way of engaging with the world.

This challenge to embodied cognition raises several further questions. For example, when we are aware of interacting with a non-embodied (yet, arguably, occasionally perceived as highly intelligent) agent without needing to recruit our skills of embodied cognition, what does it do to our own sense of agency? Is our own sense of agency still deeply grounded in our sense of embodiment in these interactions? Do we *feel* ourselves to be cognitive agents in these communications *without* feeling ourselves to be embodied agents? Or is there a peculiar dissonance where there is deliberate suspension of the recruitment of the lived body in understanding the other as a social agent while being fully aware of one's own agency (and self) as deeply embodied? In this latter scenario, could one claim that there is a widening gulf between self and the other, a gulf that is usually breached by embodied cognition of the lived body (i.e. the embodied other as a locus of experience just like I am) but embodied cognition is of no avail in these cases? It is beyond the scope of the present paper to adequately address these questions^8^ and our main goal in the paper is to bring forth these questions that emerge in the context of interaction between humans as embodied cognitive agents and technology that leans strongly toward non-embodied cognitive agency.

Furthermore, even if one is digitally interacting with an embodied agent, e.g. a human, there are major phenomenological differences in how the embodied interactions unfold in digital media in comparison with how they unfold in a non-digital face-to-face scenario. Some of these differences may even be interpreted as challenges in establishing smooth social interactions. In face-to-face, non-digital interactions with other people, embodied interaction usually proceeds as a fluid, often implicit, meaning-making process that structures the interaction. For example, you are describing the pain in your body to your doctor face-to-face and her facial expressions may give away clues about the seriousness of a possible illness which in turn may structure the communicative episode. Yet, given that embodied cognition skills often are implicit (e.g. Gallagher, 2005), you may not be aware that you are acting on the basis of embodied cues. But the fluidity of the communicative episode is structured by embodied skills of interaction. However, interaction via digital media does not necessarily guarantee such seamless embodied meaning-making that may scaffold the exchange of information, even when the other agent is a human and is perceived on a screen. In a recent paper, Willatt and Flores (2022) summarize some of the challenges that one may face in digital communication with other people from the viewpoint of embodied cognition. Examples include body fragmentation and disproportion whereby only body parts such as faces are focused on along with different proportions depending on the size of the screen, lack of eye contact, audiovisual interruptions, and discomfort in one's own body from the static postures (e.g. sitting in front of a screen) held over long periods of time. These disruptions significantly affect the real-time, seamless and often implicit meaning-making role of the lived body in agentive interactions even when the interaction is between two humans who can see each other. These practical and technical challenges are able to disrupt fluid embodied interaction via digital media in ways that are not common in face-to-face, non-digital interactions. In view of these challenges, it is reasonable to contend that the role of embodied cognition in our everyday interaction via digital media, even when the interacting partner is a human who is perceived on a screen, may be modified in comparison with non-digital face-to-face interactions. While this does not constitute a definitive argument against ECA it does provide reasonable support to make the claim that due to possible modifications and occasional reduction of real-time perceptuo-motoric feedback the lived body may not be present in digital interactions in the same way that it is present in face-to-face interactions.

Thus, the fluidity of embodied agentive skills that characterize non-digital face-to-face interactions may be either absent or modified and occasionally disrupted in interactions via digital media irrespective of whether the interaction partner is an artificial agent or a human agent. Yet, our interaction with artificial agents and human agents generally proceeds successfully via digital media. Does this challenge embodied cognitive agency (ECA) as the de facto mode of robust interaction and engagement with the world? Are ontogenetically primary embodied ways of agentive interaction being increasingly suspended in our rapidly digitalising world and is this in turn reinforcing non-embodied cognitive agency (NECA) as the de facto mode of interaction?

A prima facie answer to these questions is: yes. In interaction contexts via digital media we can, and do, interact successfully^9^ with other agents with little or no embodied engagement. In view of the above discussions, we would like to propose the following hypotheses.

1. *The hypothesis of cognitive expense (HCE):* Embodied ways of engaging with other agents is a cognitively cheap, to a certain extent automatic,^10^ real-time meaning-making process that paves the way for fluid communication. In its absence or significantly reduced presence, successful agentive interaction requires more conceptual and inferential ways of understanding others. The hypothesis of cognitive expense (HCE) states that interacting with other agents via digital media recruits more high-level cognitive resources such as inference and theoretical understanding, arguably some of which must override ontogenetically primary psychological mechanisms of embodied cognition.^11^ HCE gains support from—(i) our acceptance of artificial agents who may be present in the interaction as fairly non-embodied agents, and (ii) in our ability to keep digital communication on track even when embodied cues of interaction are disrupted. For example, when talking to another human agent via digital media, if the other person's video freezes midway through an emotionally-charged discussion then we lose access to real-time perception of their facial expressions that we, via our embodied cognitive capacities, could have grasped as expressing their mental states. But we are still able to carry on the communication without the real-time perception of facial expressions. Here we may have to heavily rely on inferential mechanisms of mindreading to infer their mental states from what they are saying. This is a much more cognitively expensive way of structuring the agentive interaction. An implication of HCE is that if there is a breakdown of the interaction then we may be more inclined to suspend the interaction in digital cases than in face-to-face non-digital cases. It is well-established in psychological studies that humans follow a “law of least mental effort” (Balle, 2002). This means that we are always on the look-out for the least cognitively expensive way of accomplishing a task. Thus, cognitively expensive resources tend to be suspended more easily if faced with challenges. For example, if we are inferring another agent's emotional state when interacting with them via digital media, as opposed to perceptually accessing their mental states in a face-to-face non-digital context, it could be the case that if the interaction gets difficult we may simply abandon inferring the other's mental state. Typically, it is embodied interaction that provides a cognitively cheaper epistemic route to another's mental states and if this route is not available then what could substitute it as a cognitively cheap interaction-repairing mechanism? We leave the question open for now.
2. *The hypothesis of disembodied agency (HDA):* When interacting via digital media with another agent (human or not), it is intuitively clear that we interact *as if* our interaction partner is a cognitive agent, i.e. an agent capable of cognitive interactions such as answering questions. Yet, as discussed in preceding sections, there may be ascription of agency and acceptance of these agents as our interacting partners without directly drawing upon embodied cognition. In fact, one may ascribe robust cognitive agency to non-embodied agents such as AI bots. This leads us to the hypothesis of disembodied agency (HDA). The hypothesis of disembodied agency states that interacting via digital media leads to the ascription of various degrees of “personhood”^12^ and agency to our interaction partners as mental beings but not necessarily as embodied beings. HDA comes in degrees. For example, when we are interacting with a loved one via digital media it is reasonable to claim that we ascribe personhood and agency to our interaction partner as both a cognitive and an embodied agent just as we would if we were meeting in a face-to-face non-digital context. Our “person model” (Newen, 2015) of the interacting agent in this case is a rich psycho-physical complexity combining both embodied and mental properties. Compare this to cases of fairly simple chatbots that do not have any visualizations. Here it is unlikely that we have a rich psycho-physical person model of the interacting agent. Yet, for the interaction to start and proceed at all there must be an ascription of a basic *as if* mental or cognitive agency. This is a case of ascription of cognitive agency without any ascription of embodied agency. The scenarios get more complicated for our commonsense ascription of agency if we are interacting with highly complex digital entities, for example AI programs that can hold meaningful philosophical conversations, write impressive articles or make artwork, yet lack embodiment in any significant sense. There may be ascription of rich mental agency in these cases, but these mental agents are not embodied agents in any meaningful way. Do these cases of disembodied agency refute the view that embodied cognition is the de facto way of engaging with the world? We propose that the answer is not a straightforward yes or no. While disembodied agency at some of its best, e.g. in cases of ascription of mental agency to highly sophisticated non-embodied AI programs, poses a serious challenge to embodied cognition, it also has its drawbacks that may eventually lead to a rethink of the importance of embodied cognitive agency. The reason is twofold—(i) the hypothesis of disembodied agency relies on the hypothesis of cognitive expense. It is cognitively demanding in various ways to consistently ascribe rich mental agency to a non-embodied agent while embodied cognition often offers a cheap and quick route for agentive interaction and (ii) disembodied agency may lead to a peculiar distortion of our own sense of the lived body by consistently and extensively suppressing our motoric engagement with an interaction partner in cognitively complex contexts. This in the long run may have a counterproductive effect on our agentive interactions and engagement with the world in general. We elaborate these claims in §3.2.

To summarize the discussion so far, to situate the debate between NECA and ECA in the context of digital interaction, we started §2 by exploring the critical role of the body in our everyday experience. We focused on a crucial insight from phenomenological discussions of embodiment, namely, that multi-agent interaction and social cognition is fundamentally built on our embodied capacities of multisensory integration. Then we proceeded to discuss the implication of this insight in the context of digital interaction and explored how humans as embodied cognitive agents interact with other agents in a digital context. We argued that in interaction contexts via digital media we can, and do, interact successfully with other agents with little or no embodied engagement. We developed the discussions further by proposing the hypothesis of cognitive expense (HCE) and the hypothesis of disembodied agency (HDA). These hypotheses further clarify the implications of interacting successfully with other agents with little or no embodied engagement in the context of digital interaction. We claimed that while HDA does pose a conceptual challenge to ECA (embodied cognitive agency) and thereby signals a win for NECA, HDA may not be the best possible alternative to ECA in the long run.

In the following section, we briefly explore a defense of embodied cognition in digital interaction from the domain of language and linguistic communication. The reason we choose to explore this domain is the following: It is reasonable to maintain that when we ascribe only mental agency to an interaction partner in the absence of any relevant feedback about the agent's embodied presence in the interaction, then the ascription of mental agency may wax and wane. For example, in our communication with AI bots, our ascription of cognitive agency and acceptance of the bot as an interaction partner may be robust when the communication is meaningful or going according to our expectations and it may be weak when it is not as meaningful as we expect it to be. We may experience moments of ambiguity when we are not sure if our disembodied interaction partner is a capable cognitive agent. In such cases a determining factor for continuing the ascription of cognitive agency to continue the interaction is the quality of linguistic communication. But what if language itself is a deeply embodied skill such that linguistic communication is fundamentally a type of embodied cognition? So here is another complication brought about by our digital interactions in the debate between non-embodied cognitive agency (NECA) and embodied cognitive agency (ECA). Digital interactions support a hypothesis of disembodied agency (HDA) that challenges embodied cognitive agency (ECA). Yet for a smooth deployment of HDA, particularly in ambiguous cases, there is the need to focus on linguistic communication, and language may itself be a deeply embodied skill. But one can take the debate even further. One may contend that the *way* that language becomes a tool of interaction in digital media does not necessarily, directly recruit language as an embodied skill. Cases of linguistic chatbots like ChatGPT illustrate the claim at some of its strongest. In the following section we briefly develop the discussion with focus on language to bring out how digital technology is importantly contributing to make the ongoing debate between NECA and ECA increasingly complicated.

## 3 Embodiment, language and the digital

A main way of agentive interaction in digital media is via language, from simple cases such as texting to more complex interaction such as social media and complex AI programs. Language is also an area where embodied cognition claims to structure fundamental meaning-making (e.g. Pelkey, 2023). From the late 1970s onwards, with the gradual development of the interdisciplinary embodied cognition movement, a growing number of theories have been arguing for the view that meaning including linguistic meaning is importantly “embodied” (e.g. Gibson, 1979; Lakoff, 1987; Lakoff and Johnson, 1999; Johnson, 2017). Thus one could claim that in spite of all that we have said in the preceding section embodied cognition does continue to play a fundamental, de facto role in digital interaction because —(i) language continues to be a main mode of digital interaction and (ii) language is fundamentally an embodied skill. Let us explore this claim in detail.

Language as fundamentally an embodied skill has been discussed in various ways in interdisciplinary contexts. The view of language as an embodied skill has been developed and elaborated by authors such as Lakoff (1987), Barsalou (2003, 2008), Bergen (2012), and Johnson (2017). These authors propose various theories about the role of embodiment in the development and everyday use of language. These range from theories about how we come to learn and use meanings of parts of speeches, for example verbs (Bergen, 2012), to how we engage in abstract conceptualizations and reasoning. These views stress the importance of an agent's sensorimotor engagement in learning and using language. Again, authors such as Johnson (2017) defend language as a robust embodied skill by arguing for a strong connection between emotions as bodily feelings and meaning.

While the above theories engage with the role of sensorimotor interaction in the acquisition and comprehension of linguistic meaning generally, the role of embodiment in language is also explored in a more direct, modality-specific context, namely in the practice of reading and the practice of handwriting. The practice of reading and the practice of handwriting are considered fundamentally deeply embodied skills in the practice of language. The role of the lived body in these cases allows a strong defense of embodied cognitive agency (ECA) and the question arises—what happens to these deeply embodied linguistic practices once digital technology enters the picture?

The visual process of reading, by which we mean an understanding of the *meaning* of perceived letters, incorporates much of what we discussed in §2 about multisensory integration and involvement of motor cognition in meaningful perception. For example, as early as in 1874 Wernicke writes, “The concept of the word “bell,” for example, is formed by the associated memory images of visual, tactile and auditory perceptions. These memory images represent the essential characteristic features of the object, bell” (Wernicke, 1874/1977, p. 117). Again In the 1960s, the noted neuropsychologist, Alexander Luria investigated the role of eye movements in meaningful perception including reading. He demonstrated that neuropathological patients with an inability to recognize visual scenes, including written words, can be helped by re-establishing the connection between motor movements usually used to explore the scene or the word.^13^ The meaning of the perceived linguistic symbols was only given once the patient was able to re-engage with the visually presented word via bodily or sensorimotor interactions. What is the implication of reading as a deep sensorimotor engagement for our present purposes?

In the debate between NECA and ECA as it plays out in the context of digital technology, a main challenge presented by reading is the following. Human agents grasp the meaning of visual symbols by drawing in real time upon a rich sensorimotor base built up by situatedness and interaction in a cultural context. But disembodied interaction partners such as AI bots decode symbols, e.g. via machine learning and large language models, with no real-time sensorimotor interaction with a context. This adds another layer of complexity to the debate about whether even sophisticated information-processing systems have only syntax and “real” understanding is out of their reach (Searle, 1980). For our present purposes, the challenge that is presented from ECA to disembodied agents in digital interaction in the context of reading is a type of asymmetry to be overcome for successful interaction. Human comprehension of perceived linguistic symbols unfolds in real-time sensorimotor interaction with the world but disembodied agents' decoding of symbols are devoid of such real-time sensorimotor interactions. For example, Nathan (2023) writes, “In psychology, Glenberg and Robertson (2000) found that human readers judge the sensibility of sentences based on the sensorimotor affordances invoked by the actions described in the sentences, rather than their lexical interconnections in high-dimensional spaces, as modeled by dAI [disembodied AI] systems widely applied in education areas such as automated essay grading (LSA; Burgess and Lund, 1997; Landauer and Dumais, 1997).” Nathan goes on to describe “the embodiment turn” in AI that aims to address the asymmetry and make responsible decisions. For our present purposes this highlights—(i) the importance of sensorimotor engagement in at least one central area in the practice of language and the possible lack thereof in some contexts of digital interaction, and (ii) the asymmetry between reading as sensorimotor engagement and reading as passive decoding of symbols that has the potential to be seriously damaging in at least some cases, for example, when disembodied agents are in charge of evaluating human educational outcomes.

In a further defense of ECA from the view of language as essentially an embodied skill, Sejnowski (2023) discusses the crucial role of embodiment in fine-tuning large language models (LLM) underlying artificial linguistic agents. Sejnowksi argues that a necessary step toward artificial agents achieving “artificial general autonomy” is incorporating LLMs into sensorimotor systems. Such incorporation shall make these agents more adaptable to the versatile nature of human language and enable them to perform a wide range of human natural language tasks. Sejnowski writes, “*LLMs need a body*. Building a body that is as flexible and adaptable as ours is even more difficult than learning how to talk….Walking and talking have much in common: generating and smoothly concatenating sequences of movements shaped by goals. LLMs can talk the talk, but can they walk the walk?” (Sejnowski, 2023). While we share the concerns raised by Nathan (2023) and Sejnowski (2023) regarding disembodied agents that are not yet capable of fully grasping the nuances of human natural language but are being put in charge of crucial tasks such as evaluating learning outcomes, we would also like to propose that there is another way to look at the challenge of, let us say, transitioning from embodied cognitive agency to the realm of the digital. This is to consider it as moving from one way of practicing a skill to another way of practicing a skill, or even practicing a different new skill. Let us elaborate the point by considering the role of embodiment in language in another more direct, modality-specific context, namely in the practice of handwriting. The practice of handwriting provides one of the strongest arguments in favor of language as an embodied skill and thereby in favor of ECA. In the following section we discuss the implications of this fundamentally embodied practice of linguistic meaning-making in the context of digital interaction where the scope of practicing this embodied linguistic skill may be significantly reduced. The question then arises - does moving on to technology that limits embodied participation necessarily lead to a deterioration in the meaning-making act? We shall argue that it is not necessarily the case that new technology leads to a deterioration in the meaning-making act. We build our claim on the philosopher Ludwig Wittgenstein's (1889–1951) views on the advancement of technology along with examples from Wittgenstein's own practice of moving from handwriting to typewriting in the course of writing his philosophical works.

### 3.1 Handwriting and the digital

Handwriting is a fundamentally, deeply embodied practice in language. Handwriting is a unique form of self-expression and identity^14^ as almost no two people have exactly the same handwriting. At the same time, handwriting is a deeply and utterly embodied practice. One may also argue that the role of the body in handwriting is not that of a mechanical vehicle for the creation of marks. Rather, by robustly engaging the sensorimotor system and interconnected processes of attention and cognition, embodiment plays a central, constitutive meaning-making role in handwriting (e.g. Perez Alonso, 2015). Language as deeply embodied through the hand-written word allows extensive exploration via traditional writing crafts such as calligraphy that not only count as linguistic activity but also as forms of art. For example, Lin and Chen (2014) explore how semantics is embedded in the visual representations in traditional Chinese calligraphy. A striking feature of the calligraphy they discuss is how the force with which a character is written embodies emotions the writer wishes to express. The authors note that the emotion words or marks reveal traces of erasing and revision, indicative of the writer's ambivalence as is natural with complex emotions, whereas the more “objective” parts do not reveal such corrections and ambivalence.

But what happens when we transition to more digital ways of writing that do not involve typical handwriting-type motoric engagement? Does it reveal that an essentially embodied way of meaning-making cannot be carried over to the digital and thus we lose a crucial form of embodied cognitive agency that expresses our individuality? Or, more dramatically, are we simply incapable of certain meaning-making acts if we suppress our handwriting and transition to keypads? Could we still create complex and subtle meanings that we can create, for example, in the case of the calligraphy mentioned above?

To discuss the issue further let us look at a concrete case of a complex text that contains both handwriting and type-writing, and is also the subject-matter of complex digitalising projects. This is the case of the *Nachlass* of the philosopher Wittgenstein (1889–1951) that he left behind upon his death. The *Nachlass* of some 20,000 pages consists of both handwritten manuscripts and typescripts.^15^

The handwritten manuscripts contain several markings that may not be directly transitioned to writing via a keyboard. To understand Wittgenstein's handwritten manuscripts in their philosophical complexities requires understanding how Wittgenstein's thinking process unfolded in real-time with his embodied engagement in the form of handwriting. Handwriting permits Wittgenstein the full material flexibility that he finds is required for pinning down the question he is pondering, finding the right approach of movement in thought and in words to approach it, and, if successful, to move it forward to a satisfactory treatment.

Wittgenstein's different handwriting modes range from sketchy, rapid, careless to well-done and calligraphic writing, from pause-less to long pauses, from fast to slow writing, from short and elliptic sentences to long sustained and even page-long paragraphs with changes in rhythm, from passion to passionless and “dead” writing showing a lack of motoric energy, from nervous to elevated and controlled writing, from “slapdash” (Wittgenstein, 2009, xi-§234) to more neat writing. For Wittgenstein himself and for Wittgenstein researchers, the handwritten manuscripts additionally speak through their dimension of tactility, reminding one of Merleau-Ponty's claim of the inseparability of the modalities of touch and perception in meaning-making.

Given how central handwriting was to Wittgenstein for the development of his work on philosophical clarifications, the question is what happens to his practice of meaning-making when Wittgenstein moves from handwriting to typing on a typewriter? How does Wittgenstein respond to this transition?

It seems no typewriting can compete with handwriting's expressivity and flexibility and several features of the handwritten manuscripts are not transferable into the typescripts, and eventually into digital documents that are machine readable. The typewriter makes each and every stroke alike, imposing a mechanical uniformity that seems to wipe out all possibility of expressing moods and subjective states through these strokes. Thus, it seems, a tour of Wittgenstein's *Nachlass* reveals that the deep and rich embodied agency that permeates Wittgenstein's philosophical discourse and comes through the pages of Wittgenstein's handwritten material, in all its materiality, is simply not reproducible in a typescript, and eventually in a digital, machine-readable format. Thus one could conclude that the absence of the deeply embodied practice of handwriting, such as in the case of typewriting and eventually producing machine-readable digital documents, leads to a deterioration in the meaning-making act.

However, Wittgenstein himself would disagree with the claim that progressing to tool use that reduces direct embodied engagement necessarily leads to a deterioration in meaning-making acts. He presents a different perspective on the advancement of technology and the role of embodiment in meaning-making than, for example, we find in Martin Heidegger's (1889–1976) and Oswald Spengler's (1880–1936) philosophy. The discussions in this context build up on wider discussions about civilization and cultural development. Heidegger famously defended the hand and the capacity to produce handwritten words as “the essence of man” and contended that “Mechanical writing…degrades the word to a means of communication” (Heidegger, 1942–43, p, 80–81). Spengler in his book *The Decline of the West* (1920; Engl. Edition, Spengler, 1927) argues that all cultures, like living organisms, pass through the stages of birth, maturation, and death. In this light, the transition from handwriting to typescripting manifests itself as part of the alienation that the human undergoes by giving more and more into the—admittedly unavoidable—technological development and thus becoming *civilization* rather than culture.

However, Wittgenstein offers ways to counter Heidegger's and Spengler's essentialist approaches to meaning-making. Writing is, according to Wittgenstein, a family of different forms of writing, and the concept of writing is a family resemblance concept (Wittgenstein, 2009, §66), with none of the specific forms of writing being paradigmatic or a prototype of meaning-making. Arguing against Spengler, Wittgenstein contends that rather than presenting one part of cultural development as the ideal, and another as its deterioration, Spengler should better have treated them in terms of neutral family resemblance.^16^

**Figure 1:**
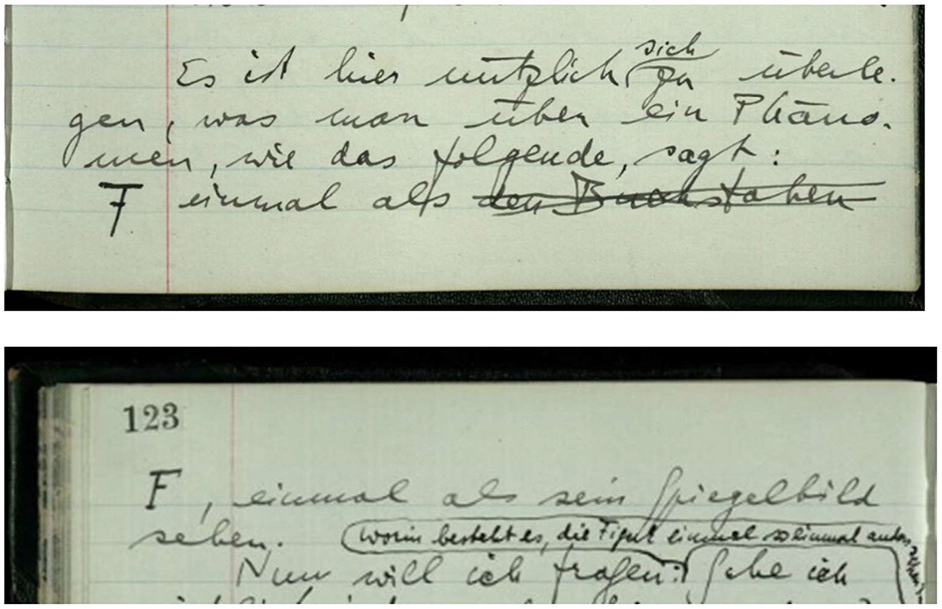
Wittgenstein Nachlass Ms-129, p. 122–123 (Wittgenstein, 2015, http://wittgensteinsource.org/BFE/Ms-129,122_f and http://wittgensteinsource.org/BFE/Ms-129,123_f). Reproduced with the kind permission of The Master and Fellows of Trinity College, Cambridge; The University of Bergen, Bergen. CC BY-NC 4.0.

**Figure 2:**
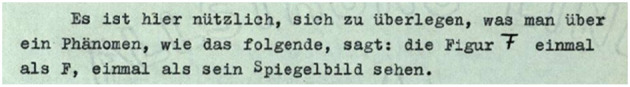
Wittgenstein Nachlass Ts-230a, p. 17 (Wittgenstein, 2015, http://wittgensteinsource.org/BFE/Ts-230a,17_f). Reproduced with the kind permission of The Master and Fellows of Trinity College, Cambridge; The University of Bergen, Bergen. CC BY-NC 4.0.

**Figure 3:**
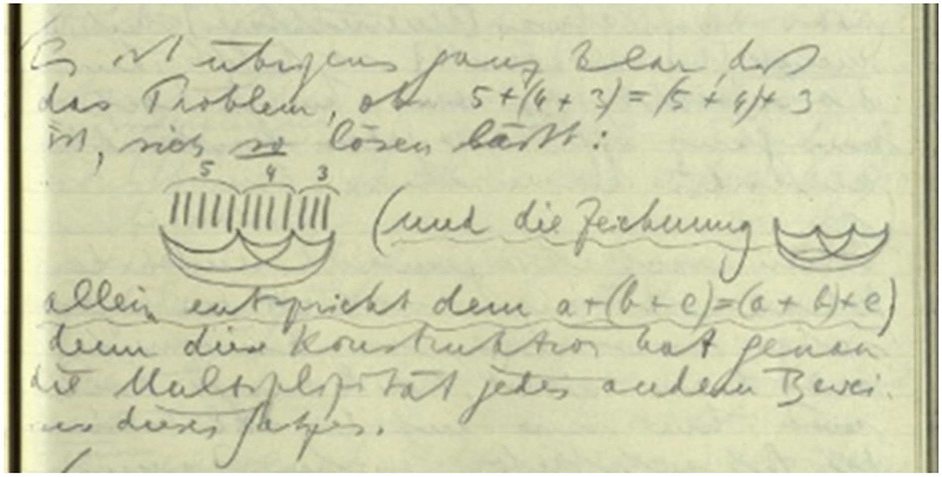
Wittgenstein Nachlass Ms-111, p. 159 (Wittgenstein, 2015, http://wittgensteinsource.org/BFE/Ms-111,159_f). Reproduced with the kind permission of The Master and Fellows of Trinity College, Cambridge; The University of Bergen, Bergen. CC BY-NC 4.0.

**Figure 4:**
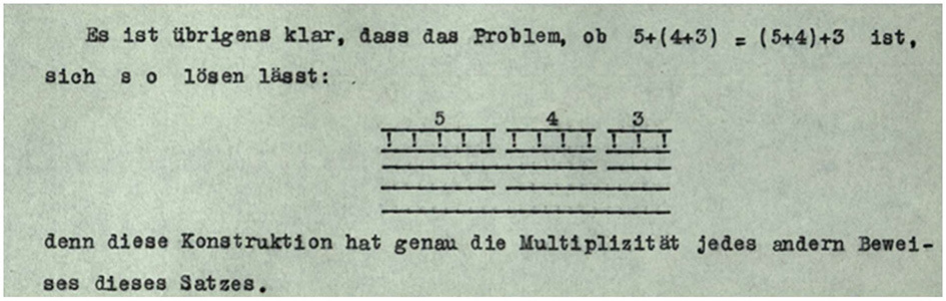
Wittgenstein Nachlass Ts-211, p. 100 (Wittgenstein, 2015, http://wittgensteinsource.org/BFE/Ts-211,100_f). Reproduced with the kind permission of The Master and Fellows of Trinity College, Cambridge; The University of Bergen, Bergen. CC BY-NC 4.0.

Consequently, the above narrative about Wittgenstein's transition from the manuscript to the typescript doesn't necessarily end with a statement of loss. The typewriter's limited expressivity and flexibility led Wittgenstein to develop a new, but simpler “markup” in the typescript. This not only applies to substitutes for various types of underlining (solid line, spacing, broken line) and the marking of text alternatives, but also to logical and mathematical notation where he had to develop substitutes for characters that the typewriter couldn't produce. Notably, this also extends to graphics. While it is true that Wittgenstein in the typescript often makes up for deficiencies in expressivity and flexibility by adding graphics in hand (see, for example, Figures 1, 2), it is also the case that Wittgenstein in response to the transition from handwriting to typewriting in the typescript often creates a strikingly simple, yet equally, if not more, clear and surveyable representation of the graphic (see, for example, Figures 3, 4).

Thus, it would be wrong to describe Wittgenstein's shift from handwriting to typescripting as necessarily involving a deterioration or diminishment from an existing, essentialist standard of meaning-making. While the medium does affect how Wittgenstein interacts with it to create meaning, the transition is more accurately described as a move from one skilled practice to another skilled practice. However, the move does not happen in a vacuum in the sense that there is total abandonment or uselessness of the first set of skills as one transitions to interact in a different way.

Consequently, according to Wittgenstein, the introduction of technology that reduces direct embodied engagement in a meaning-making act is not a straightforward argumentation for the deterioration of meaning-making. Rather it points to two developments—(i) skills from the previous way of practicing the meaning-making act may be carried over to the new way of meaning-making (e.g. Figures 3, 4) and (ii) the new technology opens up new ways of interaction and meaning-making that may not have been possible in the previous way of interaction (see Nyíri, 1994). These new ways might even find their way back into the previous way of meaning-making (see Pichler, 1994, p. 99).

Thus, moving away from a deeply embodied way of meaning-making, even in cases where the role of embodiment is completely central to the meaning-making, does not necessarily imply a deterioration in the meaning-making act. Technology that restricts or even makes redundant the role of embodied cognitive agency in meaning-making, nonetheless may require carrying over embodied practices and also open up new ways of interaction and creation of meaning.

In line with the above discussion, in the following we briefly explore a final development that can further complicate the debate between NECA and ECA. We propose that indeed the successful integration and use of digital technology *should* allow for a transfer of deeply embodied skills although such integration and use also challenge the very notion of the body itself. We clarify the reasons for our normative claim by bringing forth a final complication in the debate between NECA and ECA that we consider in this paper—a complication once again ushered in by digital technology.

### 3.2 The changing body?

The above discussions lead us to a final complication in the debate between NECA and ECA that we consider in this paper. In line with our discussions of the two hypotheses proposed in §2.1, namely, the hypothesis of cognitive expense (HCE) and the hypothesis of disembodied agency (HDA) as well as Wittgenstein's ideas of carrying over existing ways of meaning-making to new technological interfaces, we claim that the successful integration and use of digital technology *should* allow for a transfer of deeply embodied skills. The reason for the normative claim is twofold. First, deeply embodied skills of agent-environment interaction offer a cognitively cheap means of structuring and continuing communication and meaning-making. Second, embodied cognition preserves our sense of the lived body that fundamentally grounds our sense of agency in an experiential unity. Moreover, as the preceding discussions show, integration of technology that is fundamentally built on a notion of separation between cognition and embodiment does not necessarily imply acceptance of NECA over ECA. But does this imply that ECA wins over NECA? As a reply to this question, we propose that the picture is further complicated because the integration and use of digital technology may challenge the very notion of the body itself. To briefly elaborate the claim, let us return to a question we raised in the earlier parts of the paper—if digital technology's takeover of our everyday lives is challenging our embodied ways of interacting with the world and performing cognitive tasks, then what is replacing embodied cognitive agency in scenarios where it was once dominant?

The answer to the above question points in the direction of new types of embodied cognitive agency that may come into play. We have noted that reducing cognitive load in interaction by deploying embodied ways of interacting makes for better and efficient interaction. This intuition is not lost on digital technology developers who in recent years have shown significant interest in designing communicating interfaces where embodied engagement is reintroduced, but mostly through virtual embodiments^17^. Also, consider once again the case study of speech neuroprotheses by Metzger et al. (2023) that we briefly mentioned in footnote 4 as an example of extreme non-embodied cognition. But here, too, the ultimate goal of the neuroprostheses is to “...restore full, embodied communication to people living with severe paralysis” (Metzger et al., 2023) although such embodiment is very different from the original embodiment of the person and is instantiated in a facial-avatar animation. Thus, the case-study also supports the intuition that ultimately efficient interaction demands embodied interaction but the embodiment in this case is not the usual material body. The intuition of new types of embodiment has also led some philosophers to propose new conceptual frameworks for understanding these emerging new types of embodied presence, e.g. Metzinger (2018) proposes the idea of “amnestic re-embodiment” and the emergence of a “virtual *Lebenswelt*.” These bodies are not material bodies, so to speak. They are virtual reenactments of our sensorimotor skills of interaction, occasionally with augmented capacities that are impossible to recreate in our material bodies. One is tempted to make the suggestion that the hypothesis of disembodied agency (HDA) that we have proposed in this paper, lends itself toward an ascription of some sort of “super mind” and it is only intuitively fitting that if such a mind were to be connected to a body then in some way it needs to be a “super body” but not necessarily a material body. Again, authors like Gangopadhyay and Pichler (2021) have pointed out the sense of endlessness that may accompany agentive activities like meaning-making in digital media. Considering the case of creating digital texts, they write, “The potentially on-going characteristic and open-endedness of the meaning-making act in texting is greatly augmented in a digital platform and enables an author's/user's sense of agency as a participant in a vast agentive structure. The repertoires of the contents of digital media, for example, the Internet, provide at least in theory greater durability through time than physical repertoires such as paper. This may generate in agents a particularly strong sense of agency as potentially creating meaning that may last indefinitely, at least in theory. As the meaning-making joint action is not constrained by temporality, the participating agent's sense of agency may be strengthened as the creator of virtually timeless content.” (Gangopadhyay and Pichler, 2021, p. 18). It is not a far leap of imagination to couple such a sense of agency to extended senses of embodiment. If following authors like Chalmers (2022) one claims that virtual reality is genuine reality, our proposal implies that even in this new reality primarily built on mental agentative skills of engagement we nonetheless continue to be in a body, albeit of a new kind but with deep echoes of the original. In other words, with extended minds come extended bodies. Thus, as long as one has tasks to perform by engaging with the world, it is reasonable to claim that mind and body would have to function as a continuum, although technology may reveal aspects of the mind-body continuum that are hitherto unexplored.

## 4 Conclusion

In this paper we have revisited the ongoing interdisciplinary debate between two groups of theories. First, theories that contend that an agent's cognitive engagement can be defined and specified without necessarily drawing upon the agent's embodiment in any significant way. We termed this the view of “non-embodied cognitive agency” (NECA). Second, theories that claim that an agent's cognitive engagement significantly draws on the agent's bodily capacities of interaction both in terms of ontogenesis of a cognitive skill and in terms of real-time deployment of the skill. We called this the view of “embodied cognitive agency” (ECA). Our purpose in this paper is to explore how the debate between NECA and ECA gets increasingly complicated in the context of our interaction with digital technology. Thus, a central question we have discussed is how to best think of *our* agentive engagement i.e. the agentive engagement of embodied beings, in the context of interaction with digital devices that are built on a fundamental conceptual separation of cognition and embodiment. We have explored the question—does the steady development of digital technologies in our daily lives challenge the view that embodied agency is the de facto way of robust agentive engagement with the world, including with other people? We argued that in interaction contexts via digital media we can, and do, interact successfully with other agents with little or no embodied engagement. However, the picture is complicated by the hypothesis of cognitive expense (HCE) and the hypothesis of disembodied agency (HDA). HCE states that interacting with other agents via digital media recruits more high-level cognitive resources such as inference and theoretical understanding, arguably some of which must override ontogenetically primary psychological mechanisms of embodied cognition. HDA states that interacting via digital media leads to the ascription of various degrees of personhood and agency to our interaction partners as mental beings but not necessarily as embodied beings. We have argued that while HDA does pose a conceptual challenge to embodied cognition, it may not be the best possible alternative to embodied cognition in the long term.

We went on to consider a further complication in the debate between NECA and ECA, namely that digital interactions support the hypothesis of disembodied agency (HDA) that challenges ECA but for a smooth deployment of HDA, particularly in ambiguous cases, there is the need to focus on linguistic communication. Here the picture is complicated by the view that language may itself be a deeply embodied skill. We developed the discussions in this context by focusing on deeply embodied linguistic practices such as reading and handwriting. Taking the cue from the discussions on handwriting, we presented a Wittgensteinian perspective of viewing technological development as not necessarily a deterioration in a meaning-making act but rather as a movement from one set of skilled activity to another set of skilled activity. Finally, we concluded with the claim that even if the hypothesis of disembodied agency (HDA) favors NECA over ECA, the successful integration and use of digital technology should allow for a transfer of deeply embodied skills in view of cognitive load and preservation of the experiential unity that grounds our sense of agency. However, existing in new realities created by digital interactions may imply that our embodiment is also changing to adapt to these realities.

## Data Availability

The original contributions presented in the study are included in the article/supplementary material, further inquiries can be directed to the corresponding author.

## Footnotes

^1^See Madary (2022) for a different view.

^2^Developmental psychology abounds with examples of children's expectations of successful social interactions via embodied expressions (e.g. Rochat, 2009; Reddy, 2010) and also what happens when those expectations are not met (e.g. Weinberg et al., 2008).

^3^In a recent article, in the context of use of AI chatbots in healthcare, Altamimi et al. (2023) write, “While AI chatbots offer many benefits, it is critical to understand their limitations. Currently, AI lacks the capacity to demonstrate empathy, intuition, and the years of experience that medical professionals bring to the table…. These human traits are invaluable in effective patient care, especially when nuanced language interpretation and non-verbal cues come into play.” In the context of our present discussions, it is important to note that even in the case of sophisticated AI chatbots the lack of embodied engagement presents a challenge in patient care.

^4^An extreme case of non-embodied cognition in interaction with a digital device is the case of speech neuroprotheses recently described by Metzger et al. (2023). The authors directly recorded neural activity in a patient with severe limb and vocal paralysis when the patient tried to “silently speak sentences” (Metzger et al., 2023). The authors write, “Neural activity was processed and used to train deep-learning models to predict phone probabilities, speech-sound features and articulatory gestures” (Metzger et al., 2023). However, such cases of non-embodied cognition are at present extremely rare in our daily interaction with digital devices.

^5^By “subjectivity” we mean being the subject of experience.

^6^Whether these agents actually have intentionality or not is a different matter. For example, humans ascribe intentionality to even simple geometric shapes when these are presented as interacting in a certain way (e.g. Scholl and Tremoulet, 2000).

^7^We deliberately use the rather vague term “others” here to indicate that the “other” may be a human agent or an artificial agent with or without a human-like appearance.

^8^Each of these questions may well be thought of as an interdisciplinary research topic in itself. Thus without attempting to provide answers to these questions in this paper, we simply aim to chart how the debate between ECA and NECA may unfold if situated in the eventually inevitable context of digital technology.

^9^Here one may ask: does interacting “successfully” imply interacting meaningfully? This is an interesting question and is beyond the scope of the present paper. For our present purposes, we use “successfully” in a rather loose pragmatic sense of the term to indicate that we are able to perform tasks by interacting with these agents. Although we cannot go into detail in the present paper about meaningfulness in the context of artificial agents, we would like to point out that there is developing interdisciplinary discussions about the explanatory viability of the classical distinction between the syntax and semantics of language in the context of artificial linguistic agents (e.g. Peregrin, 2021). We thank an anonymous reviewer for raising the issue.

^10^E.g. Gallese and Goldman, 1998.

^11^It may be objected that learning to interact with other agents via digital media may over time reduce the cognitive load with practice. But this need not necessarily be the case because inferences and drawing on a theory-theory style understanding of other minds (e.g. Gopnik and Wellman, 1992) will always require more top-down psychological and neural resources than perceptual understanding of other minds (e.g. Gallagher, 2008; Zahavi, 2011) and embodied skills of agentive interaction. Perceptual understanding of other minds and embodied skills of interaction may not be directly recruited in digital agentive interaction.

^12^The concept of personhood has a history of rich theoretical debates in philosophical literature, yet the concept lacks clear “theoretical definiteness” (Moran, 2009). For our present purposes, we shall use the concept simply to mean a commonsensical or folk psychological attribution of qualities of a person to an entity. This may include describing the entity in psychological terms such as “thinking”, “wanting” and so. It may well extend to include moral and ethical attributions. For example, a viciously trolling AI bot may be attributed the property of being evil. For our present purposes it suffices to accept that we attribute psychological and moral qualities to entities without going into the details of what qualities we attribute. We are open to an idiosyncratic ascription of personhood. We are not interested in the present paper in metaphysical discussions for ascriptions of personhood or in questions about whether the entity actually has psychological states. We focus simply on folk psychological ascriptions for purposes of interaction.

^13^A patient that Luria treated was able to write down sentences dictated to him but was unable to copy the sentences when presented in printed form. Luria asked the patient to trace the letters with his fingers and follow the tracing patterns with his eyes. After some practice the patient could identify the letters without any finger-movement and only by tracing eye movements. Eventually the patient could recognize the letter without the tracing eye movement (Luria, 1963).

^14^Consider the case of signatures.

^15^See https://wab.uib.no/.

^16^“Spengler could be better understood if he said: I am *comparing* different periods of culture with the lives of families; within the family there is a family resemblance, while you will also find a resemblance between members of different families; family resemblance differs from the other sort of resemblance in such & such ways etc. What I mean is: We have to be told the object of comparison, the object from which this approach is derived, so that prejudices do not constantly slip into the discussion. Because then we shall willy nilly ascribe what is true holds of the prototype of the approach of the comparison to the object to which we are applying the approach as well; & we claim “it *must always be*...” (Wittgenstein, 1998, p.21).

^17^The metaverse is the future of digital connection | Meta.
